# Integrative Analysis of DNA Methylation and microRNA Expression Reveals Mechanisms of Racial Heterogeneity in Hepatocellular Carcinoma

**DOI:** 10.3389/fgene.2021.708326

**Published:** 2021-09-07

**Authors:** Rency S. Varghese, Megan E. Barefoot, Sidharth Jain, Yifan Chen, Yunxi Zhang, Amber Alley, Alexander H. Kroemer, Mahlet G. Tadesse, Deepak Kumar, Zaki A. Sherif, Habtom W. Ressom

**Affiliations:** ^1^Department of Oncology, Lombardi Comprehensive Cancer Center, Georgetown University, Washington, DC, United States; ^2^MedStar Georgetown University Hospital, Washington, DC, United States; ^3^Department of Mathematics and Statistics, Georgetown University, Washington, DC, United States; ^4^Department of Pharmaceutical Sciences, North Carolina Central University, Durham, NC, United States; ^5^Department of Biochemistry and Molecular Biology, College of Medicine, Howard University, Washington, DC, United States

**Keywords:** epigenomic and transcriptomic data, DNA methylation, miRNA expression, cancer disparities, hepatocellular carcinoma

## Abstract

Pathologic alterations in epigenetic regulation have long been considered a hallmark of many cancers, including hepatocellular carcinoma (HCC). In a healthy individual, the relationship between DNA methylation and microRNA (miRNA) expression maintains a fine balance; however, disruptions in this harmony can aid in the genesis of cancer or the propagation of existing cancers. The balance between DNA methylation and microRNA expression and its potential disturbance in HCC can vary by race. There is emerging evidence linking epigenetic events including DNA methylation and miRNA expression to cancer disparities. In this paper, we evaluate the epigenetic mechanisms of racial heterogenity in HCC through an integrated analysis of DNA methylation, miRNA, and combined regulation of gene expression. Specifically, we generated DNA methylation, mRNA-seq, and miRNA-seq data through the analysis of tumor and adjacent non-tumor liver tissues from African Americans (AA) and European Americans (EA) with HCC. Using mixed ANOVA, we identified cytosine-phosphate-guanine (CpG) sites, mRNAs, and miRNAs that are significantly altered in HCC vs. adjacent non-tumor tissue in a race-specific manner. We observed that the methylome was drastically changed in EA with a significantly larger number of differentially methylated and differentially expressed genes than in AA. On the other hand, the miRNA expression was altered to a larger extent in AA than in EA. Pathway analysis functionally linked epigenetic regulation in EA to processes involved in immune cell maturation, inflammation, and vascular remodeling. In contrast, cellular proliferation, metabolism, and growth pathways are found to predominate in AA as a result of this epigenetic analysis. Furthermore, through integrative analysis, we identified significantly differentially expressed genes in HCC with disparate epigenetic regulation, associated with changes in miRNA expression for AA and DNA methylation for EA.

## Introduction

Hepatocellular carcinoma (HCC) is the third leading cause of cancer-related mortality worldwide. The incidence is higher in African-Americans (AA) and is associated with more advanced tumor stage at diagnosis and lower survival rate compared to other racial groups including European-Americans (EA) (Ryerson et al., 2016; Islami et al., 2017; American Cancer Society [ACS], 2018). A population-based, retrospective cohort study investigated racial disparities in HCC based on the SEER database from 2000 to 2012. It was reported that AA were more likely to be diagnosed with advanced HCC stages, but less likely to receive cancer-directed surgery treatments compared with EA (Franco et al., 2018). Although racial health inequalities are mostly attributed to socioeconomic differences, there is a growing realization that genetic and epigenetic events can also contribute to cancer incidence and clinical outcomes (Wallace et al., 2011; Daly and Olopade, 2015). For example, there is emerging evidence linking epigenetic events including DNA methylation and microRNA (miRNA) expression to cancer disparities in breast, prostate, endometrial and colon cancers (Mehrotra et al., 2004; Kwabi-Addo et al., 2010; Wang et al., 2012, 2016; Long et al., 2013; Nwaneri et al., 2016; Hashimoto et al., 2017; Shiina et al., 2017).

Among the myriad of epigenetic changes that can occur in human cancers, dysregulation of DNA methylation and miRNAs are frequent events in HCC. Compelling evidence over the years has demonstrated that both DNA methylation and miRNAs are important in modulating cellular transformation and tumorigenesis. Patterns of global hypomethylation and regional hypermethylation are characteristic changes demonstrated in HCC that can play a role in processes contributing to neoplastic transformation including, activation of oncogenes, silencing of tumor suppressor genes and chromosomal instability (Ehrlich, 2009). Likewise, miRNAs constitute important epigenetic markers that regulate carcinogenesis by acting post-transcriptionally on mRNAs, contributing to the progression of HCC (Nasr et al., 2019). Epigenetic changes are thought to occur early in carcinogenesis and may be amenable to detecting HCC with low tumor burden, including early detection, minimal residual disease, and relapse (Nishida and Goel, 2011; Zhang, 2015).

While epigenetic changes to the methylome and miRNome associated with HCC have been characterized independently, few studies have explored the integrated relationship between these two forms of epigenetic regulation. Among differentially methylated CpG sites in HCC, Song M. A. et al. (2013) found 10,775 located within or adjacent to gene promoters using the Illumina 450k array. Of these CpG sites, 493 are associated with miRNA genes, suggesting that methylation at these sites is responsible for miRNA regulation. Using the same array platform, Shen et al. (2013) reported 28,017 CpG sites (5.8%) to be hypermethylated in HCC. This indicates that miRNA genes are likely to be targeted by DNA hypermethylation in HCC and additional studies are needed to elucidate the relevance of this phenomenon. Through monitoring the hypermethylation and resultant inactivation of miRNA genes, HCC can be distinguished from benign liver tumors; this hypermethylation also correlates with poor prognosis among HCC patients, representing a promising new diagnostic measure and prognostic marker for HCC. *MiR-122* is among many unique and well-studied dysregulated miRNAs that is highly expressed in the human liver and expression levels have been shown to correlate with DNA methylation levels at the promoter in HCC (Song K. et al., 2013). Aberrant DNA methylation of miRNA genes can also affect several signaling pathways important in hepatocarcinogenesis and is used for maintenance of cancer stem cell phenotype (Anwar and Lehmann, 2014). Another study integrated miRNAs along with their mRNA targets and their methylation profile into a regulatory meth-miRNA–mRNA network using a systems biology approach (Zahid et al., 2019).

In addition to being targets in epigenetic regulation, miRNAs can directly target other epigenetic factors, such as DNA methyltransferases or methylation-related proteins, which can regulate chromatin structure. Genome-wide approaches have comprehensively identified dysregulated miRNAs in HCC tumor tissues compared to non-tumor tissues (Borel et al., 2012). For example, *miR-29c-3p* has been demonstrated to target DNMT3B and altered expression of this miRNA in HCC has resulted in aberrant methylation of key tumor suppressors (Wu et al., 2019). There is a strong connection between the methylome and miRNome, and any dysregulation of this complex system can result in various physiological and pathological conditions (Piletic and Kunej, 2016).

The balance between DNA methylation and miRNA expression and its potential disturbance in HCC can vary by race. There have been reports linking epigenetic events including DNA methylation and miRNA expression to cancer disparities (Suzuki et al., 2012; Anwar and Lehmann, 2014). Several studies have reported coordinated actions between miRNAs and other epigenetic mechanisms to reinforce the regulation of gene expression using The Cancer Genome Atlas (TCGA) data and other datasets (Anwar and Lehmann, 2014; Bianchi et al., 2017). An integrated analysis of multi-platform data of HCC using TCGA- Liver Hepatocellular Carcinoma (LIHC) data examined DNA methylation, mRNA expression, miRNA expression and protein expression in 196 patients to understand the molecular landscape of HCCs. Integrated analysis enabled the development of a p53 target gene expression signature correlating with poor survival and several potential therapeutic targets (Cancer Genome Atlas Research Network, 2017). The TCGA study also reported that hepatitis C virus (HCV) infection was significantly higher in EA and AA compared to Asians and in patients with cirrhosis (Cancer Genome Atlas Research Network, 2017).

In this paper, we aim to explore potential crosstalk between two types of epigenetic regulation in HCC through integrated analysis of DNA methylation and miRNA expression data. Furthermore, we seek to identify race-specific differences that may shed light on previously unknown mechanisms contributing to racial disparities in HCC by investigating the mutual regulation of DNA methylation and miRNA expression on gene expression (Barefoot et al., 2019; Varghese et al., 2020a,b).

## Materials and Methods

### Characteristics of the Study Population

Table 1 provides the characteristics of 16 HCC patients whose cancerous and paired non-tumor liver tissues were analyzed to acquire DNA methylation, miRNA-seq, and mRNA-seq data. The patients for this study were recruited at MedStar Georgetown University Hospital (MGUH) through an inclusion protocol approved by the Georgetown University institutional review board (IRB). All subjects provided informed consent forms and Health Insurance Portability and Accountability Act (HIPAA) authorization forms. The patients were diagnosed to have HCC based on well-established diagnostic imaging criteria and histology. Tissues were donated by patients scheduled for elective surgical procedures due to the cancerous or potentially cancerous conditions and/or other related benign conditions. Based on a review by a pathologist, all tumor and adjacent non-tumor tissues are non-cirrhotic. There was no significant difference between the two racial groups with respect to age, gender, and other clinical covariates.

**TABLE 1:** Characteristics of study population.

		**AA (*N* = 8)**	**EA (*N* = 8)**
Age	*Mean (SD)*	59.4 (12.8)	65.6 (11.9)
Gender	*Male*	78.6%	62.5%
BMI	*Mean (SD)*	31.3 (10.9)	28.4 (15.0)
HCV serology	*HCV Ab+*	57.1%	31.2%
	*HCV RNA+*	21.4%	25.0%
HBV serology	*anti HBC+*	7.1%	0.0%
	*HBs Ag+*	28.6%	0.0%
Smoking	*Yes*	64.3%	50.0%
	*No*	35.7%	50.0%
Alcohol	*Yes*	42.9%	43.8%
	*No*	57.1%	56.3%
AFP	*Median (IQR)*	11.2 (147.3)	5.4 (14.025)
AST	*Median (IQR)*	132 (140)	127.5 (171.8)
ALT	*Median (IQR)*	141 (135)	101 (128.5)
Child Pugh score	*Mean (SD)*	6.2 (1.7)	6.4 (1.3)
	*Median (IQR)*	6 (1)	6 (1.25)
Child Pugh class	*A*	78.6%	62.50%
	*B*	7.1%	37.50%
	*C*	7.1%	0%
HCC stage	*Stage I*	42.9%	37.5%
	*Stage II*	21.4%	25.0%

### DNA Methylation Analysis

DNA samples from 32 (16 tumor and 16 adjacent non-tumor) fresh-frozen tissues were extracted using the QIAamp DNA mini kit (QIAGEN) and bisulfite was converted using the EZ DNA Methylation-Gold Kit (Zymo Research). Genome-wide DNA methylation analysis was performed using the Infinium HumanMethylationEPIC BeadChip (Illumina) that covers more than 850,000 CpG sites at single-base resolution. All procedures were conducted according to the manufacturer’s instructions. Raw signal intensity data were imported from idat files into the ChAMP package in R/Bioconductor for processing. Preprocessing of the data included quality control and normalization steps. All 16 paired liver tissues in this study passed quality control. Out of the 865,918 probes, we filtered out many including those in X and Y chromosomes, non-CpG probes, probes mapped within 10 bp of a common SNP or within 15 bp of a repetitive element, and multi-hit probes. Normalized β-values representing the remaining 731,852 probes were converted to *M*-values for subsequent analysis (Barefoot et al., 2019).

### miRNA-Seq and mRNA-Seq Profiling

RNA samples from the 32 fresh-frozen samples were isolated using the RNeasy Plus Universal Mini Kit (Qiagen) with on-column RNase-free DNase treatment. The RNA quality and quantity were estimated based on ultraviolet-visible (UV-VIS) spectrophotometry using the NanoDrop ND-1000 spectrophotometer. The RNA integrity was assessed using the Agilent RNA 6000 Nano Kit on the Agilent 2100 Bioanalyzer.

For miRNA expression profiling, aliquots of the RNA samples were analyzed using the Qiagen’s QIAseq miRNA library kit for library preparation prior to sequencing by Illumina NextSeq 550 platform using 2 × 150 bp paired-end (PE150). The miRNA-seq data were analyzed using the QIAseq miRNA quantification data analysis software. First, we calculated the unique molecular index (UMI) counts and primary miRNA mapping. Then, the UMI counts were analyzed to calculate the changes in miRNA expression. Finally, the quantified data were normalized using the trimmed mean of *M*-values (TMM) method before statistical analysis was performed.

For mRNA expression profiling, aliquots of the RNA samples described above were indexed using the TruSeq RNA Access Library Prep Kit prior to sequencing by Illumina HiSeq 4000 using 150 bp pair end (PE150). The mRNA-seq data contained an average of 33M reads per sample. The fastq files were imported into Partek Flow for quality assessment, alignment, and estimating transcript abundance. Alignment was performed using the spliced transcripts alignment (STAR) algorithm. The aligned reads were quantified to the transcriptome through Expectation Maximization (E/M) method implemented in Partek Flow and normalized using the TMM method.

### Mixed-ANOVA Model

A mixed-ANOVA model was applied separately to all three omic datasets to find the significant differentially methylated CpG sites (DMCs) or probes as well as differentially expressed (DE) miRNAs and mRNAs. The two-way mixed-ANOVA model used for analysis is shown in Eq. (1):

(1) $$y_{ijk}=\mu +\tau_{i}+\alpha_{j}+\beta_{k}+\gamma_{jk}+\varepsilon_{ijk}$$

$$i=1..n_{jk};j=1,2;k=1,2;\varepsilon_{ijk}∼N\left(0,\sigma^{2}\right);\tau_{i}∼N\left(0,\omega^{2}\right)$$

where μ is the grand mean; τ_*i*_ are the random patient effects; α_*j*_ and β_*k*_ are the fixed main effects for the *j*^th^ race and *k*^th^ disease, respectively, γ_*jk*_ is the interaction of *j*^th^ race effect and *k*^*th*^ disease effect; *j* = 1, 2 denotes AA and EA, respectively, *k* = 1, 2 indicates tumor and adjacent non-tumor tissue, respectively. For race-specific analysis, we considered different contrasts. To compare tumor vs. adjacent non-tumor for AA, we test, (β_1_ + γ_11_) − (β_2_ + γ_12_) = 0. Similarly, (β_1_ + γ_21_) − (β_2_ + γ_22_) = 0 is tested to compare tumor vs. adjacent non-tumor for EA. The hypothesis test for the main effect of disease (over all levels of the race factor) is $\left(\beta_{1}-\beta_{2}\right)+\frac{1}{2\left(\gamma_{11}-\gamma_{12}\right)}+\frac{1}{2\left(\gamma_{21}-\gamma_{22}\right)}=0.$For multiple testing correction, we use the Benjamini-Hochberg (BH) false discovery rate (FDR). Fold change (FC) values are calculated considering the median of raw intensities values for mRNA-seq and miRNA-seq data. For DNA methylation data, the beta difference between HCC and adjacent non-tumor is used in lieu of FC.

### Integrative Analysis

**FIGURE 1:**
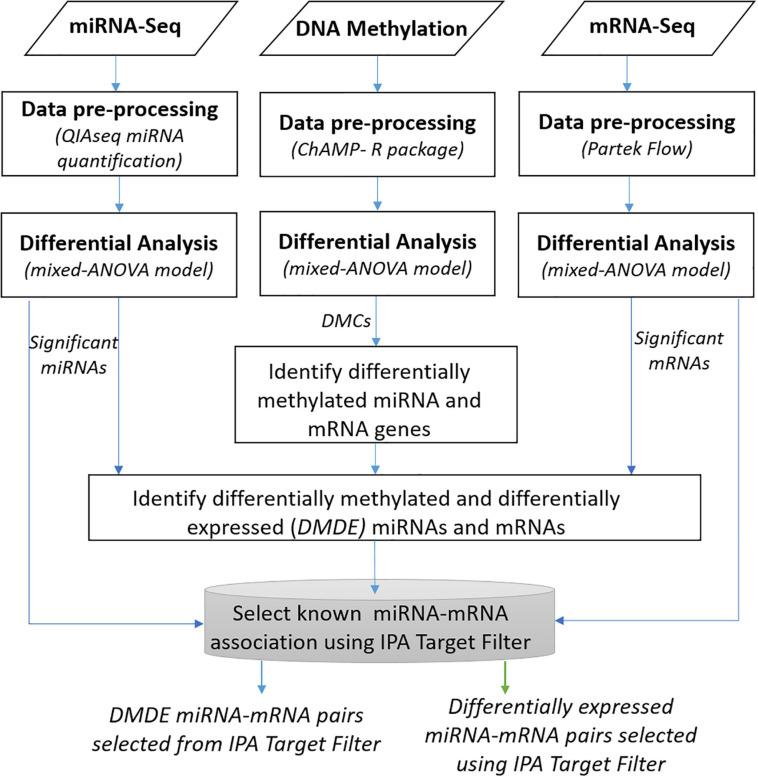
Overview of integrative analysis of multi-omic data.

Figure 1 depicts a workflow for integrative analysis of DNA methylation, miRNA-seq, and mRNA-seq data. We conducted multiple integrated analyses to explore crosstalk between the methylome and miRNome in HCC. To evaluate the potential regulation of miRNAs by DNA methylation, we investigated miRNAs regulated by DNA methylation and resultant changes in gene expression from differential miRNA regulation. To identify any potential influence of DNA methylation and miRNA on specific mRNA targets, we generated a list of selected mRNAs whose expression patterns differed significantly in tumor vs. non-tumor tissues from the mixed-ANOVA model. Then, we matched the expression data with differentially methylated CpG sites located in the promoter regions to identify DMDE genes. Likewise, we identified potential miRNA gene targets from select molecules found to have significant differences in expression between tumor and non-tumor tissues. Specifically, the miRNAs with FDR < 0.05 were matched to target mRNA pairs from databases using the IPA miRNA target filter function that provides biological effects of miRNAs based on experimentally validated interactions from TarBase and miRecords, as well as high confidence predicted miRNA-mRNA interactions from TargetScan and the Ingenuity Knowledge Base. The experimentally verified highly confident miRNA targets were then overlapped with the list of significant mRNAs from the mRNA-seq analysis to select reported targets of the significant miRNAs.

To explore mechanisms of racial heterogeneity in HCC whereby gene dysregulation is brought about by varying epigenetic changes in AA compared to EA, we analyzed whether DE mRNA targets of DE miRNA in AA overlap with genes that are differentially methylated and differentially expressed (DMDE) in EA. We investigated genes that were exclusively found to be regulated by miRNA in AA (that were not methylated) in which the reverse was found to be true to EA, i.e., genes that were regulated by methylation only (not miRNA). We specifically looked at this combination for comparison because of the heightened levels of differential methylation identified in EA and contrasting heightened levels of differential miRNA expression in AA.

### Pathway Analysis

Pathway analysis was performed to investigate possible interactions amongst epigenetically regulated genes in HCC. Analysis was done individually at each omics level as well as at various stages of integration. Pathway analysis was performed on differentially expressed miRNAs and gene targets to explore processes regulated by microRNAs in HCC (Supplementary Table 2C). Separate analysis was performed on differentially expressed and differentially methylated genes to look at overlapping and distinct processes regulated by DNA methylation as compared to miRNA (Supplementary Table 2B). Then, combined analysis of all epigenetically driven pathways described as “epi-pathways” in HCC was performed including reciprocally regulated DMDE genes, DE miRNAs, and DE miRNA-mRNA target pairs. This epi-pathway analysis was performed on all HCC cases as well as separately on cases from AA and EA [Supplementary Table 2A (ALL), Supplementary Table 4A (AA), and Supplementary Table 4B (EA)]. Epi-pathway analysis was also performed on paired tumor tissues with adjacent normal non-tumor tissues from the TCGA LIHC data collection [Supplementary Table 6A (ALL), Supplementary Table 6B (AA), and Supplementary Table 6C (EA)]. Qiagen’s Ingenuity Pathway Analysis (IPA) tool was used to perform all pathway analysis.

## Results

### Analysis of Individual Omics Data by Mixed-ANOVA Model

Table 2 presents the significant molecules identified by analysis of DNA methylation, miRNA expression, and mRNA expression data independently using mixed ANOVA model. The table shows the number of differentially methylated CpG sites (DMCs), DE miRNAs, and DE mRNAs when comparing tumor vs. adjacent non-tumor tissues in AA, EA, and ALL (AA and EA combined). Venn diagrams for the significant molecules identified within each omics dataset between AA, EA, and ALL are shown in Supplementary Figure 1.

**TABLE 2:** Significant molecules identified within each omic dataset included.

	**DMCs; FDR < 0.05 and | **Δ**β | > 0.1**	**DMCs in the promoter regions of mRNAs [DM genes]**	**DMCs in the promoter regions of miRNAs [DM miRNAs]**	**DE mRNAs (FDR < 0.05)**	**DE miRNAs (FDR < 0.05)**
AA	38,751	2,394 [1,077]	49 [23]	1,765	26
EA	274,540	18,227 [5,879]	291 [110]	1,875	7
ALL	339,447	26,958 [7,118]	473 [148]	4,762	82

*DE, differential expression; DM, differential methylation; DMC, differentially methylated CpG sites.*

**FIGURE 2:**
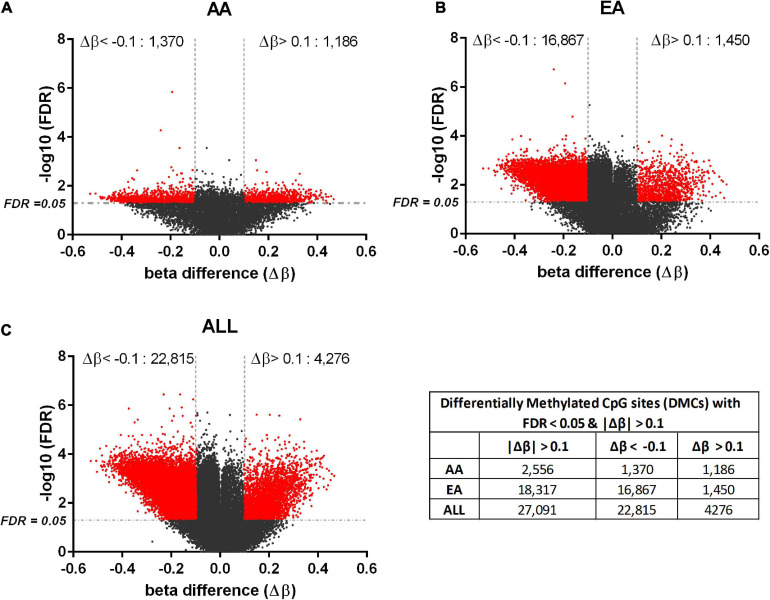
Differentially methylated CpGs in tumor vs. non-tumor tissues in AA, EA, and AA+EA (ALL) combined. Volcano plot of the significant CpGs with FDR < 0.05 and |Δβ| > 0.1 (shown in red). The methylated CpGs were identified by analyzing HCC vs. adjacent non-tumor tissues in AA **(A)**, EA **(B)**, and ALL **(C)**; The beta difference is calculated as, Δβ = [β_(HCC)_ – β _(adj. *non–tumor*)_].

For DNA methylation analysis, DMCs with FDR < 0.05 and absolute value of beta difference > 0.1 were considered significant. Hyper- and hypo- methylated CpG sites were identified in HCC compared to non-tumor tissues for AA and EA separately as well as AA and EA combined. 825 DM genes and 17 DM miRNAs were found to overlap amongst AA and EA; however, a larger number of DMCs was identified in EA (Figure 2). Supplementary Table 5 shows the number of molecules identified with reciprocal and non-reciprocal epigenetic regulation, in AA, EA, and ALL analyses.

**FIGURE 3:**
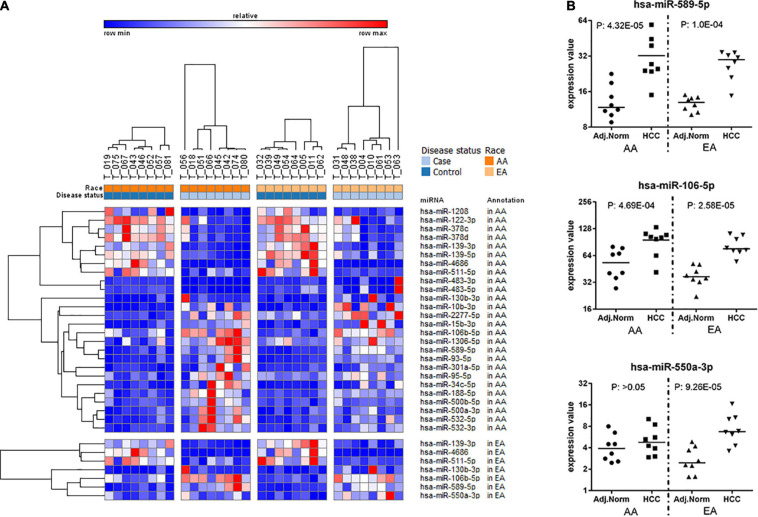
Differentially expressed miRNAs in tumor vs. non-tumor tissues in EA and AA. **(A)** Differentially expressed miRNAs comparing HCC vs. non-tumor tissues in AA (Piletic and Kunej, 2016) and EA (Mehrotra et al., 2004). **(B)** Dot plots for three selected miRNAs differentially expressed in tumor vs. non-tumor tissues in AA and EA.

Figure 3A depicts a heatmap of the significant miRNAs identified in AA and EA. Figure 3B presents dot plots for three selected miRNAs differentially expressed in tumor vs. non-tumor tissues in AA and EA. There are 26 miRNAs differentially expressed in AA and 7 miRNAs in EA; however, *hsa-miR-550a-3p* was differentially expressed only in EA. Two other miRNAs, *hsa-miR-589-5p* and *hsa-miR-106-5p* were found to be significant in both AA and EA, but *hsa-miR-589-5p* was observed to be more significant in AA and *hsa-miR-106-5p* to be significant in EA and marginally significant in AA. As shown in Table 2, despite many differences observed at the epigenetic level, there were 763 differentially expressed mRNAs overlapping between AA and EA.

Furthermore, we compared AA vs. EA within the tumor and within adjacent non-tumor tissues separately using ANOVA. For example, we examined the two differentially methylated and differentially expressed (DMDE) mRNAs (Supplementary Figure 2) that are unique to AA. We found these genes to be significantly altered in comparing tumors from AA vs. EA, not in in the comparison among the adjacent non-tumor samples between AA and EA. Similarly, considering MEF2A, CXCL8, STX11, TFEC, CEP68, and GHR that are among the DMDE mRNAs found to be unique to EA, they were found to be significantly different with *p*-value < 0.05 in comparing tumors from AA vs. EA.

### Integrative Analysis

Integrative analysis was performed to explore epigenetic regulation of gene expression in HCC. We denote DMCs located in the promoters of DE mRNAs or miRNAs as DMDE mRNAs or DMDE miRNAs, respectively. In addition, DE gene targets of DE miRNAs were identified to integrate the miRNA-seq and mRNA-seq data. The significant pairs as a result of this integrative analysis are shown in Table 3. Venn diagrams for the significant pairs identified by integrative analysis of DNA methylation, miRNA expression, and mRNA expression, between AA, EA, and ALL are shown in Supplementary Figure 2. These results point toward epigenetic changes that may be mechanistically responsible for the dysregulation of these combined DMDE mRNA and DE miRNA-target genes in tumor vs. adjacent non-tumor tissues.

**TABLE 3:** Significant pairs identified by integrative analysis of DNA methylation, miRNA expression, and mRNA expression.

	**DM genes** – DMCs in the promoter regions of genes	**DMDE genes** – DMCs in the promoter regions of DE mRNAs (reciprocally regulated)	**DM miRNA** – DMCs in the promoter regions of miRNA	**DMDE miRNA** – DMCs in the promoter regions of DE miRNA (reciprocally regulated)	**DE miRNA-mRNA targets** – DE mRNAs found to be targets of DE miRNAs (reciprocally regulated)
AA	1,077	72 (40)	23	0	206 (114)
EA	5,879	411 (219)	110	1	66 (38)
ALL	7,118	1,189 (674)	148	19 (8)	1,452 (879)

*DE, differential expression; DM, differential methylation; DMDE, differential methylation and differential expression.*

**FIGURE 4:**
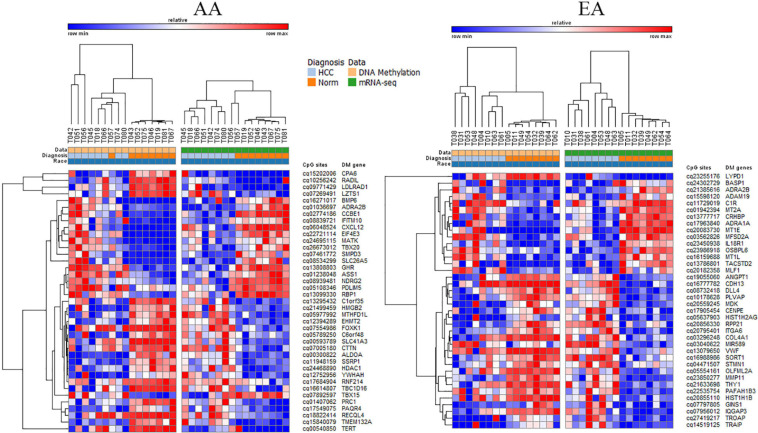
DMDE genes selected by integrative analysis of DNA methylation and mRNA or miRNA expression data. DMDE genes specific to AA (left) and specific to EA (right).

There were 23 overlapping DMDE mRNAs between AA and EA, but a significantly larger number of DMDE mRNAs were identified in EA. Likewise, there were no DMDE miRNAs identified in AA and only one DMDE miRNA (*MIR589*) was identified in EA. The heatmap in Figure 4 presents DMDE mRNAs and miRNAs identified in AA and EA. We selected the differentially methylated genes (mRNAs and miRNAs) and looked at the expression of these mRNAs and miRNAs in the mRNA-seq and miRNA-seq data, respectively. The CpGs that are in the promoter location of differentially methylated genes were cross examined for differential expression and selected those that are reciprocally regulated as shown in the figure. We also performed a Pearson correlation analysis on these selected DMDE genes and found that 67% of those in AA and 53% in EA are correlated with correlation coefficient greater than 0.5. In the analysis of the samples from EA and AA combined, we revealed differential methylation in the promoters of 16 differentially expressed miRNAs with 403 differentially expressed downstream mRNA targets.

**FIGURE 5:**
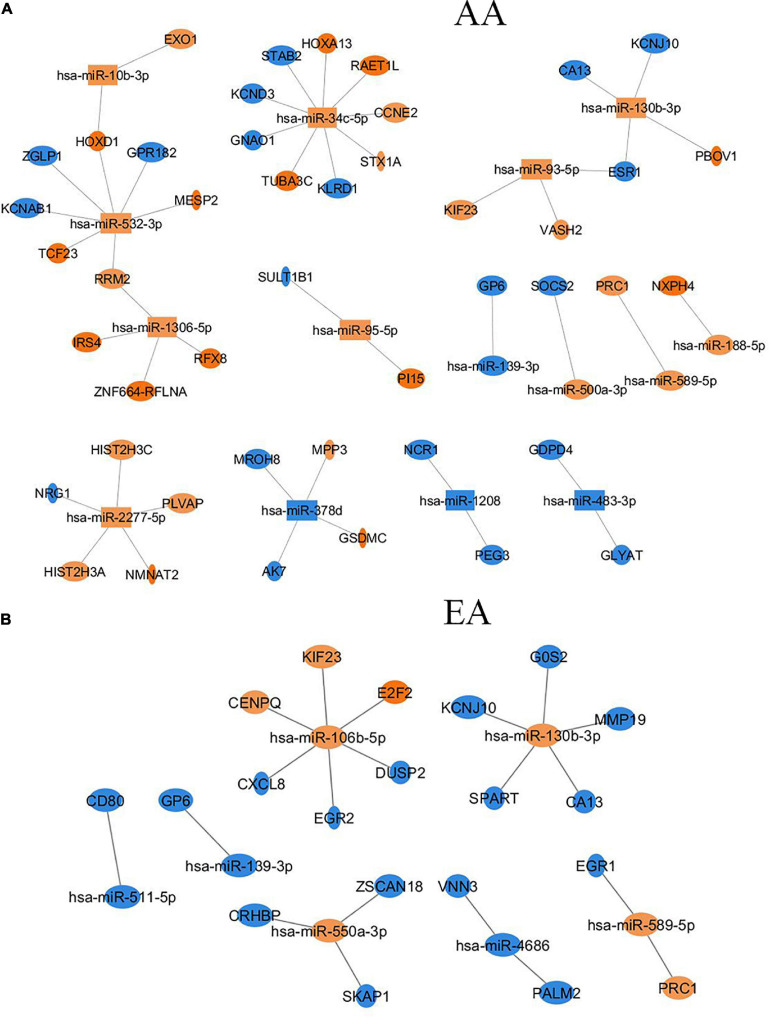
Differentially expressed miRNAs and their target mRNAs (experimentally validated or highly confident in databases) that are differentially expressed with |FC| > 2 in AA **(A)** and EA **(B)**.

**FIGURE 6:**
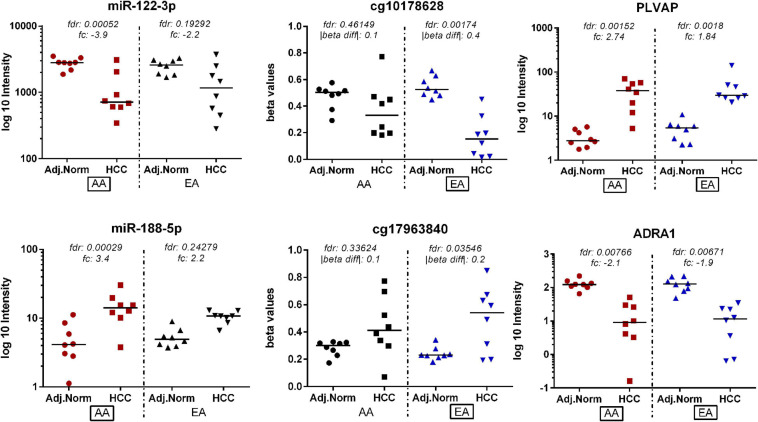
Genes identified as being potentially regulated by different epi-mechanisms in AA vs. EA. These target mRNAs are significantly up- or down- regulated in tumor vs. non-tumor tissues in both AA and EA. However, miRNA targeting these genes are differentially expressed only in AA and CpG sites located in the promoters of these genes are differentially methylated only in EA.

Similarly, there were six overlapping DE miRNAs identified between AA and EA. However, in contrast to the results found by the DNA methylation data analysis, there were a larger number of DE miRNAs identified in AA; 26 miRNAs in AA and 7 miRNAs in EA. This resulted in the downstream increase in the number of DE miRNA-mRNA targets in AA. The microRNA Target Filter analysis in IPA identified 206 mRNA targets overlapping with the differentially expressed mRNAs from our analysis. Significant mRNAs that are targets of DE miRNAs with fold change > 2 up- or down- regulation are shown in Figure 5. Only experimentally validated or highly confident targets found from the target filter analysis are shown in the figure. We also investigated whether the DE mRNA targets of DE miRNAs in AA overlap with genes that are DMDE in EA, suggesting differential epigenetic regulation at play in dysregulation of common genes involved in HCC pathogenesis. We identified 22 mRNAs that are regulated by different epi-mechanisms in AA vs. EA. Specifically, these mRNAs are significantly up- or down- regulated in tumor vs. non-tumor tissues in both AA and EA. However, 14 miRNAs targeting these genes are differentially expressed only in AA and CpG sites located in the promoters of these genes are differentially methylated only in EA. Figure 6 presents two examples of these genes with such relationship. All 14 miRNAs, the 22 mRNAs, and the CpGs are presented in Supplementary Table 1.

Additionally, we explored miRNA regulation of DNA methylation through targeting DNA methyltransferases or other epi-machinery genes including methylation-related proteins. We identified 51 predicted miRNA gene targets that are known DNA methylation machinery genes, possibly providing a means for crosstalk between DNA methylation and miRNA expression in HCC (Supplementary Table 3). In particular, *hsa-mir-101-3p* is found to be significantly downregulated miRNA in HCC and DNMT3A is identified as a gene target that is significantly upregulated in HCC from our data. Furthermore, we identified miRNAs that target other epi-machinery genes as well, such as DNA demethylase *TET2* and methyl-CpG-binding proteins, *ZBTB12* and *ZBTB21*. In addition, histone methylases and demethylases were found to be targets of differentially expressed miRNAs in HCC from this analysis, providing another role for the epigenetic regulation of HCC, which may be a topic of interest for future targeted analyses (Supplementary Table 3).

**FIGURE 7:**
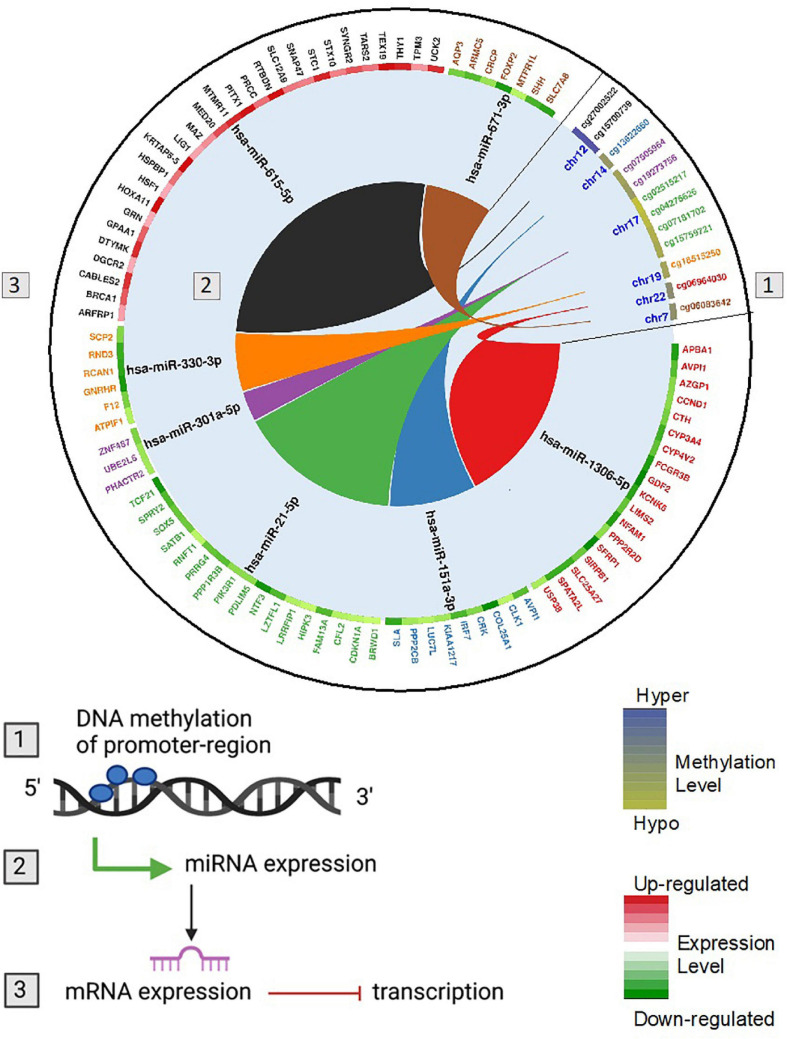
Circos plot showing DNA methylation regulation of miRNA and genes potentially targeted by miRNAs at the post-transcriptional level. The colors follow the integration across (1) DNA methylation → (2) miRNA expression → (3) target mRNA expression. Hypermethylated (blue) and hypomethylated (yellow) status of CpG sites is depicted as a heatmap for differentially methylated CpG though to regulate miRNA expression. Up (red) and down (green) regulation of gene expression is depicted as a heatmap for each gene that is an identified miRNA target. The miRNAs have reciprocal regulation compared to the target mRNA genes. For example, cg06964030 is significantly hypomethylated in HCC and located in the promoter region of hsa-miR-1306-5p. Hsa-miR-1306-5b is significantly upregulated in HCC and from using the miRNA Target Filter function in Ingenuity Pathway Analysis (IPA) is known to regulate genes APBA1, AVPI1, AZGP1, CCND1, CTH, CYP3A4, CYP4V2, FCGR3B, GDF2, KCNK6, LIMS2, NFAM1, PPP2R2D, SFRP1, SIRPB1, SLC25A27, SPATA2L, USP38. All of these genes are significantly downregulated in HCC tumors relative to adjacent normal tissues.

**FIGURE 8:**
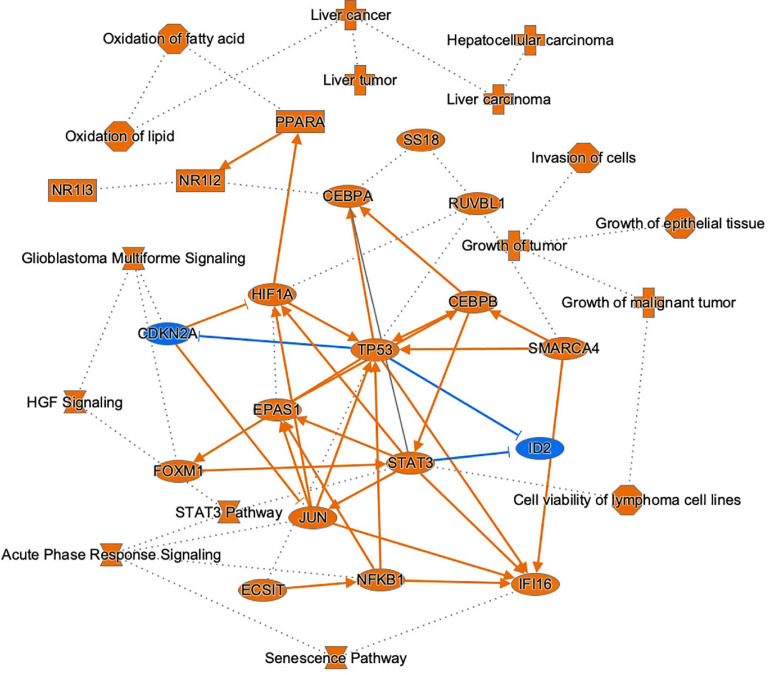
Graphical summary of molecules, pathways, and functions regulated by integrating DNAm-miRNA-mRNA interactions in HCC. This network mostly represents the highly rated interactions of genes, pathways and functions found to exhibit this form of complex regulation. Selected target genes of differentially methylated and differentially expressed miRNAs in HCC are depicted (blue = downregulated, orange = upregulated), as well as top pathways and functions associated with these molecules. A solid line represents a direct interaction between the two gene products and a dotted line means that there is an indirect interaction.

To link DNA methylation to changes in gene expression and parallel linking of DE miRNA to changes in gene expression, we conducted analysis of DNA methylation–miRNA–mRNA changes. The circos plot in Figure 7 depicts the differentially methylated CpG sites located in the promoters of differentially expressed miRNAs and the downstream differentially expressed miRNA-mRNA targets. Differential methylation of 12 CpG sites shown that are within the promoters of 7 miRNAs led to differential expression and downstream reciprocal regulation of 88 highly confident or experimentally validated miRNA-mRNA targets. Integration across DNA methylation → miRNA expression → target mRNA expression is demonstrated through color-coding as well as interaction connectors in the circos plot. Network analysis of the target mRNAs of DMDE miRNAs identified by this integrative analysis is shown in Figure 8. Among the genes shown in the figure, *TP53, JUN, STAT3, NFkB1*, and *CDKN2A* stand out as well-known tumor suppressors and oncogenes intrinsic to carcinogenesis in HCC (Martin and Dufour, 2008). These complex layers of regulation demonstrate involvement in processes critical to hepatocarcinogenesis, regulating many carcinogenic signaling pathways in HCC including growth, proliferation, and acquired malignancy of cells.

### Epi-Pathways in HCC Associated With Race

**FIGURE 9:**
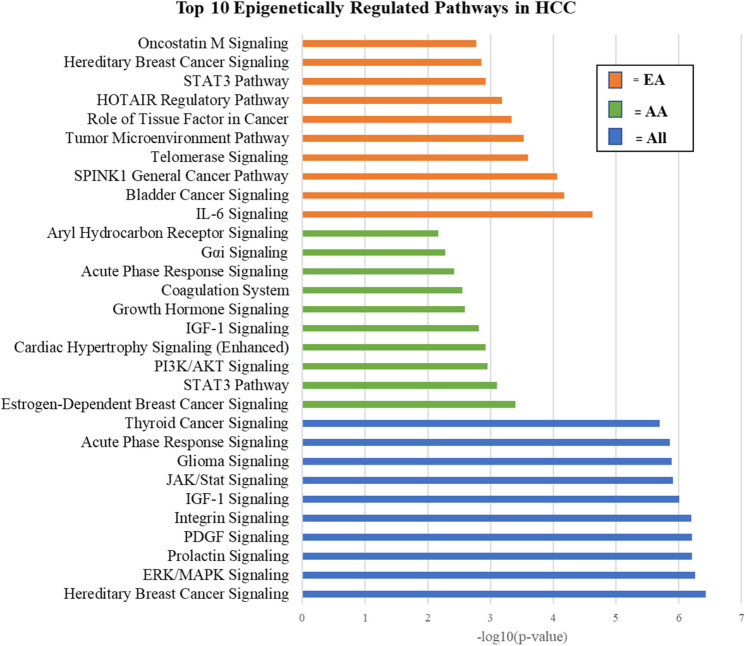
Top 10 epigenetically regulated pathways in HCC.

Pathway analysis was performed on epigenetically regulated genes identified specifically in either AA or EA. We found regulation by miRNAs to be more intrinsic in separating tumors from adjacent non-tumor tissues in AA, while changes due to differential methylation to be more abundant in EA. This distinction led to important differences in the pathways of epigenetically regulated molecules in AA and EA which may reveal biological mechanisms of racial heterogeneity in HCC. Figure 9 depicts the top 10 epigenetically regulated pathways in HCC (ALL, i.e., AA and EA combined) compared to those specific to AA and EA, respectively. We also identified top epigenetically regulated pathways and functions in HCC that are mirrored in the literature from other studies, including Aryl Hydrocarbon Receptor signaling, Senescence Pathway, cAMP-mediated signaling, PIK/AKT/mTOR, Ras/Raf/MAPK, and ESR1/ERK signaling (Bhat et al., 2018). The top 100 epigenetically regulated pathways are listed in the Supplementary Table 2A. We identified changes in DNA methylation and miRNA expression as playing a role in transcriptional regulation, cell growth and proliferation, development, EMT, and invasion in HCC (Supplementary Tables 2B,C; Puszyk et al., 2013; Toh et al., 2019).

### Analysis of TCGA Data

For further evaluation, we analyzed miRNA-seq, mRNA-seq, and DNA methylation data from The Cancer Genome Atlas (TCGA)^1^ network. While the TCGA DNA methylation data were generated using Illumina Infinium Human Methylation 450 K BeadChip (Illumina 450 K array), the data in our study were generated using the Infinium HumanMethylationEPIC BeadChip (Illumina) that covers more than 850,000 CpG sites. We considered a subset of the TCGA data obtained by analysis of tumor and adjacent non-tumor liver tissues from 7 AA and 24 EA patients from the TCGA-LIHC cohort. These patients were matched using the TCGA patient identifiers to select their corresponding non-tumor tissues. We analyzed the TCGA data using the same approach we used to analyze our data. Although the TCGA miRNA-seq data consisted of 2,588 miRNAs, there were more than 90% missing values for the 31 subjects considered and thus only 751 miRNAs were used for subsequent analysis. Of these, 707 miRNAs overlapped between the TCGA and our datasets. Supplementary Table 7 summarized the molecules identified with reciprocal and non-reciprocal epigenetic regulation in HCC.

There was significant overlap in the significant pathways obtained by analysis of epigenetically regulated molecules identified from the cohort presented in this paper compared with similar analysis conducted with data from the TCGA LIHC cohort. A striking 98% of epi-pathways were found in common when pathway analysis was performed by combining the data from AA and EA (ALL). Also, significant overlaps were found in the results of the pathway analysis parsed by race, with 95% overlap for EA and 71% overlap for AA (Supplementary Tables 6A–C).

## Discussion

Although DNA methylation plays an essential role in normal biologic processes, abnormal DNA methylation is observed as an early event in hepatocarcinogenesis. Unlike genomic alterations known to be associated with cancer, the epigenetic mechanisms driving the pathogenesis of HCC have only recently begun to be explored. The epigenome consists of diverse mechanisms of regulatory control, including those involving DNA methylation and microRNA expression. Most of these prominent forms of epigenetic regulation are thought to act in a repressive manner. Conventionally, epigenetic changes in HCC are associated with hyper methylation of the promoters leading to silencing of tumor suppressors as well as global hypo methylation changes contributing to chromatin instability. Likewise, increased expression of oncogenic microRNAs is associated with repressive targeting and gene silencing of tumor suppressor mRNA targets. In this study, we performed comprehensive profiling and integrative analysis of DNA methylation, miRNA expression, and mRNA expression in tumor tissues relative to paired adjacent non-tumor tissues.

We compared key demographic factors influencing our analysis, including race, and highlighted mechanistic similarities and differences amongst AA and EA. We observed differing changes to the epigenetic landscape of HCC in AA compared to EA. We observed several differentially methylated CpG sites in EA as compared to AA. Comparatively, the miRNA expression was altered to a larger extent in AA with a larger number of significant miRNAs detected in AA. While epigenetic processes appear to play a role in regulation of proliferation, metabolism, and growth pathways in AA, there is preferential epigenetic regulation of immune cell maturation, inflammation, and vascular remodeling in EA (Supplementary Tables 4A,B). For example, IL-6 Signaling, IL-15 Signaling, FLT3 Signaling in Hematopoietic Progenitor cells and VEGF Signaling are unique epi-pathways in HCC cases from EA. In comparison, Growth Hormone Signaling, Wnt/β-catenin Signaling, and Aryl Hydrocarbon Receptor Signaling present as unique epi-pathways in HCC cases from AA. Through investigation of the mutual regulation of DNA methylation and miRNA expression on gene expression, we identified race-specific differences that may shed light on previously unknown mechanisms contributing to racial disparities in HCC. We also identified differentially expressed genes in HCC that are regulated by miRNAs in EA and alternatively regulated by DNA methylation in AA. Differential epigenetic regulation of these genes may be a factor in racial disparities resulting in variable response to therapeutics and prognosis. These subtle differences may contribute to mechanisms of disparity influencing patient outcomes in AA vs. EA.

## Conclusion

In this study, we provided an assessment of the DNA methylation and microRNA expression profiles within neoplastic HCC tissues to engender key information in supporting the diagnosis, predicting the clinical behavior, and designing specific therapeutic plans for AA and EA racial groups. We performed comprehensive profiling and integrative analysis of DNA methylation, miRNA expression, and mRNA expression in tumor tissues relative to paired adjacent non-tumor tissues. We also conducted multiple integrated analyses to explore crosstalk between the methylome and miRNome. We identified top epigenetically regulated pathways and functions in HCC. Combining DNA methylation and miRNA data allows for a deeper dive into the complexities of epigenetic interaction and regulation. This study demonstrates the value in integrative analysis to assess mutual regulation of DNA methylation and miRNA expression in identifying mechanisms of disparity in HCC.

The interaction of DNA methylation with microRNA expression and the resultant changes to the HCC transcriptome may reveal key differences in racial groups, having the potential to transform cancer management and improve mechanistic knowledge. However, there have been few studies geared toward assessing the mechanisms of epigenetic regulation in racially diverse HCC patient populations. Whilst the mutational landscape of HCC has many commonalities amongst diverse patient populations, environmental and behavioral differences are likely to play a larger role on the epigenome and may contribute to racial disparities in HCC through previously unknown mechanisms. Particularly, several therapeutics targeting epigenetic molecules or processes may act differently in different racial groups contributing to observed efficacy differences and patient outcomes. Additional studies focused on comprehensive and integrative analysis of these complementary omics datasets are necessary to fully capture the dynamics of these interactions. Ultimately, better understanding of these mechanisms may prove useful to guide the development of efficient therapeutic approaches in racially diverse patient population. Our future work will focus on analysis of multi-omic datasets with larger sample size to explore the interactions of these epigenetic regulators with the ultimate goal being to identify and validate epigenetic changes associated with racial disparity in HCC.

## Data Availability Statement

The datasets presented in this study can be found in online repositories. The names of the repository/repositories and accession number(s) can be found below: https://www.ncbi.nlm.nih.gov/geo/, GSE176289.

## Ethics Statement

The studies involving human participants were reviewed and approved by the Georgetown University IRB. The patients/participants provided their written informed consent to participate in this study.

## Author Contributions

RV and YC performed the statistical analysis. MB performed the pathway analysis and discussed the results. RV, MB, SJ, and YZ generated the results, figures, and created discussion. AA helped analyzing the DNA methylation data. MT provided guidance on the design and development of statistical models, and reviewed the statistical methods. HR conceived the study design and outlined the manuscript organization. ZS, DK, and AK provided interpretation of results. RV and MB wrote the manuscript. All authors critically reviewed and approved the manuscript.

## Conflict of Interest

The authors declare that the research was conducted in the absence of any commercial or financial relationships that could be construed as a potential conflict of interest.

## Publisher’s Note

All claims expressed in this article are solely those of the authors and do not necessarily represent those of their affiliated organizations, or those of the publisher, the editors and the reviewers. Any product that may be evaluated in this article, or claim that may be made by its manufacturer, is not guaranteed or endorsed by the publisher.

## Footnotes

1
https://tcga-data.nci.nih.gov/tcga/

## Supplementary Material

Supplementary Figure 1 Venn diagram for the significant molecules identified within each omic dataset, between AA, EA, and ALL (AA and EA combined). DE, differential expression; DM, differential methylation; DMDE, differential methylation and differential expression.

Supplementary Figure 2 Venn diagram for the significant pairs identified by integrative analysis of DNA methylation, miRNA expression, and mRNA expression, between AA, EA, and ALL (AA and EA combined). DE, differential expression; DM, differential methylation; DMDE, differential methylation and differential expression.

Supplementary Figure 3 HGF identified as gene with disparate forms of epigenetic regulation in EA compared to AA HCC. HGF is found to be significantly regulated by miR-483-3p in tumor vs. adjacent non-tumor tissues in AA (but not EA). In contrast, significant hypomethylation of cg15567428 found in the promoter of the HGF gene was identified unique to EA. In parallel, decreased expression of miR-483-3p in AA and hypomethylation of cg15567428 in EA are found to have a similar relationship leading to a paradoxical dual-down regulatory role leading to decreased expression of the HGF gene in HCC.

Supplementary Table 1 Comprehensive list of differentially expressed genes in HCC with disparate epigenetic regulation associated with changes in miRNA expression in AA and DNA methylation in EA.

Supplementary Table 2A Top 100 epigenetically regulated pathways in HCC (reciprocally regulated DMDE genes, DE miRNA, and DE mRNA targets).

Supplementary Table 2B Top 100 methylome-associated pathways in HCC (reciprocally regulated DMDE genes).

Supplementary Table 2C Top 100 miRNome-associated pathways in HCC (DE miRNA and reciprocally regulated DE mRNA targets).

Supplementary Table 3 MiRNA regulation of DNA methylation machinery genes in HCC (ALL).

Supplementary Table 4A Top 100 epigenetically regulated pathways of HCC in AA (reciprocally regulated DMDE genes, DE miRNA, and DE mRNA targets).

Supplementary Table 4B Top 100 epigenetically regulated pathways of HCC in EA (reciprocally regulated DMDE genes, DE miRNA, and DE mRNA targets).

Supplementary Table 5 Identified molecules with reciprocal and non-reciprocal epigenetic regulation in HCC.

Supplementary Table 6A TCGA Data—Top 100 epigenetically regulated pathways in HCC (ALL)—(reciprocally regulated DMDE genes, DE miRNA, and DE mRNA targets).

Supplementary Table 6B TCGA Data- Top 100 epigenetically regulated pathways of HCC in AA (reciprocally regulated DMDE genes, DE miRNA, and DE mRNA targets).

Supplementary Table 6C TCGA Data- Top 100 epigenetically regulated pathways of HCC in EA (reciprocally regulated DMDE genes, DE miRNA, and DE mRNA targets).

Supplementary Table 7 TCGA Data—Identified molecules with reciprocal and non-reciprocal epigenetic regulation in HCC.
