# Supplementary figures and images for: Integrative Analysis of DNA Methylation and microRNA Expression Reveals Mechanisms of Racial Heterogeneity in Hepatocellular Carcinoma
Source: Front Genet. 2021 Sep 7;12:708326. doi: 10.3389/fgene.2021.708326 (PMC8453167; doi:10.3389/fgene.2021.708326)

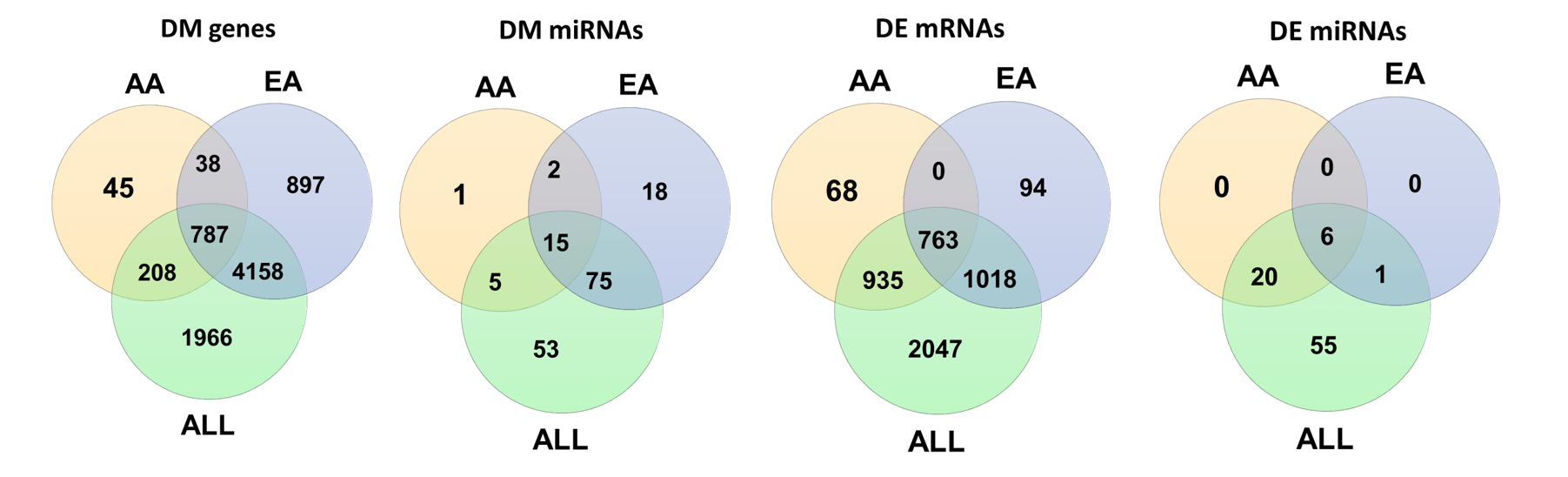

Supplement: Supplementary Figure 1 — Venn diagram for the significant molecules identified within each omic dataset, between AA, EA, and ALL (AA and EA combined). DE, differential expression; DM, differential methylation; DMDE, differential methylation and differential expression. [file Image_1.PNG]

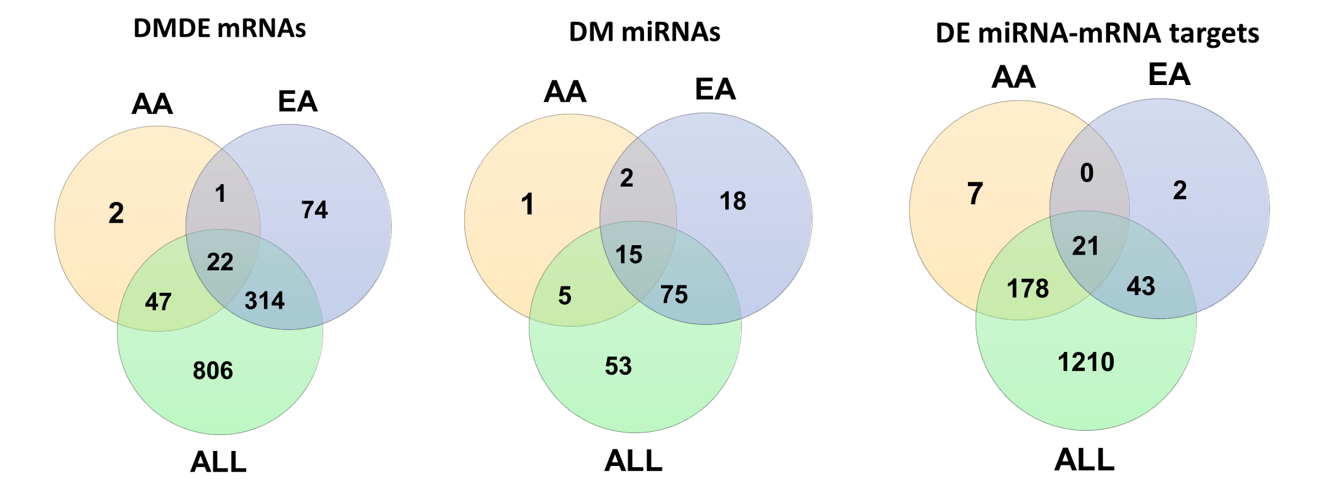

Supplement: Supplementary Figure 2 — Venn diagram for the significant pairs identified by integrative analysis of DNA methylation, miRNA expression, and mRNA expression, between AA, EA, and ALL (AA and EA combined). DE, differential expression; DM, differential methylation; DMDE, differential methylation and differential expression. [file Image_2.PNG]
